# On the Replanting of Teeth

**Published:** 1871-09

**Authors:** Geo. T. Barker


### SELECTED ARTICLES.

---

### ARTICLE IV.

### *On the Replanting of Teeth.*

By Geo. T. Barker, D. D. S.

[A Paper read before the Southern Dental Association, Charleston, April, 1871.]

Perhaps no subject at the present time is attracting more attention among the advanced thinkers in dentistry than that which is the subject of this paper: for not only does the theme open up a vast field of study and conjecture, how far and how frequently, such a mode of procedure may be practicable, but it brings us naturally to a study of the pathological changes which arise from such action, and the relation which the replanted tooth bears to the rest of the economy. We are led to study the physiological as well as the pathological changes which take place in that remarkable membrane, the periosteum—a membrane, whose agency, recently recognized, as a bone producer, can be said to have remodelled a branch of surgical practice during the last few years, having thus effected the preservation of many limbs that otherwise would have been amputated, and destroyed the usefulness, if not the life of many a patient. The subject of replanting extracted teeth is not new in dentistry; in many works of old writers we find a short reference to this subject, a summary of which may be stated as follows: If a wrong tooth has been extracted, or a tooth has been knocked out, it should be washed, the socket cleansed and the tooth replaced. Some believed that union of the separated pulp in the tooth and its former connection took place, because they found no discoloration subsequently ensue.

This view obtains to some extent at the present time, for we occasionally see such assertions in articles in dental journals, though in my judgment the position is erroneous, and cannot be supported by any reliable evidence or probable theory, for reasons which I shall present hereafter. But it is not alone in our dental works that reference has been made to this subject, for our magazines have contained from time to time articles where success is chronicled as the result of replanting extracted teeth. Generally, the authors have resorted to this mode of procedure when teeth have been knocked from their sockets by accident, and I doubt not that all within the hearing of my voice, who have been in practice for a considerable number of years, will remember such attempts in their own experience followed by varying successes. Though these cases have been recorded, the question whether or not teeth should be extracted for the removal of some obscure and otherwise—with our present knowledge—incurable disease, has not yet received the attention which it deserves, and has only been slightly referred to in brief communications, with which I am familiar. My object, however, at this time, is to direct the attention of gentlemen present to a more advanced step in this direction, and urge upon all to prosecute experiments in this particular field, viz: The extraction and replanting of teeth as a means of arresting incurable dental diseases. I will detail as an example, the following case, which has received treatment at my hands:

Miss H——, aged seventeen years, of a sanguo-bilious temperament, and decidedly healthy organization, presented herself for treatment for face and toothache, in July, 1870. She was at that time residing at Long Branch, N. J., a popular sea side resort on the Atlantic coast, and as I had made a careful examination of her teeth but a few weeks previously, which, with an unsuccessful search at that time for exposed pulps or dental irritation, led me to fly to that frequent statement of the bewildered physician and dentist, viz: that she was probably suffering from neuralgia, and had better see her physician and obtain some appropriate remedy. This request was complied with, but without relief being obtained from a pain which partook of the nature of tic douloureux, but which seemed to be most severe in the lower teeth of the right side. In a week she again presented herself, having suffered greatly. On making at this time a careful examination of her lower teeth, I found slight dental irritation at the root of the right second lower molar. This tooth had a small gold filling upon its grinding surface, and injections alternately of hot and cold water demonstrated the presence of an irritated pulp. My first step in the treatment was to remove filling, and, if possible, expose the pulp. This I could not succeed in doing, as the attempt gave so much pain. Failing in this, the cavity was filled with a pledget of cotton, saturated with carbolic acid, and covered with Hill's stopping, to prevent irritation from thermal changes. The carbolic acid was used with the object of inducing union between some of the elements of the tooth bone, as it is believed that, under favorable circumstances, carbolic acid will unite with albumen, an insoluble substance which will protect parts from irritation, and which is hence used for the treatment of sensitive dentine. Finally, blood was freely drawn from the gum over the affected root, and after the bleeding had subsided, the gum structure in the neighborhood was painted with the following preparation, which I have used in numerous instances with most favorable results:

℞.—Tr. iodinii, fl. ʒ iv;

Etheris, fl. ℥ i.—*Misce.*

The object of taking blood from the neighboring parts was to relieve the distended vessels of their accumulated contents; that of applying the ethereal preparation of iodine was to favor absorption of any effusion, the product of inflammatory action, a result which is more frequently obtained with this substance than with any other with which I am familiar, though care is necessary in its use not to allow the cheek or lips to touch the gum until the ether has evaporated, leaving the pellicle of iodine intact, or blistering of the parts will occur.

The treatment, however, was entirely unsuccessful, as the pain continued as bad as before, particularly at night. After a few days, the carbolic acid and gutta-percha filling were removed, and the ordinary arsenical paste introduced, for while it is a principle that the paste should not be introduced, until the inflammation has subsided in a pulp, as absorption will not readily take place in inflamed parts, yet occasionally the application will excite so much irritation that the pulp will die from over stimulation. In this case there was a thick covering of bone protecting the pulp from the arsenious paste, and absorption would have to take place through this plate before the pulp could be influenced. The introduction of the paste only added to the pain, and though retained for some forty-eight hours, did not in any way diminish the sensitiveness of the pulp. The carbolic acid and Hill's stopping were again introduced, only to be followed by the same result as above stated. At this time the young lady had become so much worn down from loss of sleep that resort was had to the syrup of the hydrate of chloral as an anodyne, the following prescription being used:

℞.—Chlorali hydratis, ʒ ss;

Aquæ distil., fl. ℥ iv;

Syr. aurant.,

Mucil. acaciæ, aa fl. ℥ ss.—*Misce.*

A tablespoonful was given at night. And I would here state though used very frequently for six months by this young lady, it was not found necessary to increase the dose, as a tablespoonful at night would cause a good night's rest to ensue, while no headaches or other unpleasant result were present the succeeding day. It is too much to claim that the continued use of anodyne would present the same peculiar advantage in all cases, as we must ordinarily expect to resort to an increase in quantity and frequency of doses, the longer any sedative is used.

The pulp paste was again introduced, was retained for forty-eight hours, though she suffered excruciating pain, but was finally removed, the pulp still being as senstive as ever. Resort was now had to an anæsthetic; the young lady was placed fully under the influence of chloroform, (as her friends preferred that anæsthetic, though my own preference is always for ether,) the pulp cavity was drilled into, and the living pulp wholly removed. Of course, my belief was, that the trouble would now end. The pulp chamber was left open; the gums thoroughly painted with officinal tincture of capsicum, and the patient dismissed with the assurance that all pain would in a short time cease. The next day the tooth was as painful as ever, the inflammation of the periosteal membrane as great as before, the tooth was elongated, and there was no improvement whatever over the former condition.

The following treatment was then resorted to: one-sixth of a grain of morphia was injected hypodermically over the root. This was followed by temporary relief only. Five drops of tincture of aconite root were then placed on cotton and laid over the effected root; partial relief followed. The tooth was not constantly painful, but shooting pains would occur frequently during the day; no sleep could be obtained at night without using the syrup of hydrate of chloral, as the pain was generally constant at that time. In this way the tooth continued for the space of nearly six months, every effort being made on my part to reduce inflammatory action, and to have it terminate in resolution, and every agent with which I am familliar, that could possibly be used, was tried, unsuccessfully. An effort was then made about the first of the present year, to obtain a termination of the inflammation by suppuration, in the hope that an abscess would be formed, which could be more successfully treated. Heat to the face on the effected side was tried, frequent use of hot water in the mouth, hot fomentations and cataplasms, all of no avail; it would not terminate in suppuration, but would simply ache! ache! In the whole of this time the young lady had never once asked to have the tooth extracted, but had borne the pain with a heroism truly wonderful, and which in consideration of my numerous failures entitled her to be considered the most courageous person with whom I had ever met in my professional experience. The determination was now made in my mind to extract the tooth, and if practicable, replace it. Accordingly, chloroform was administered; the tooth was extracted; the end of the root (fortunately this second molar had the roots joined,) was cut off, and the tooth was instantly placed in a solution of tepid water, fl. ʒ ss, carbolic acid, gtt. v. The socket was wiped with the solution, and the blood carefully removed. A broach was introduced into the pulp cavity, which was found be entirely free, and the tooth was carried into its former socket, the shape of which favored its retention. The cavity through the tooth was left open, and the patient was dismissed. No after treatment except an astringent wash has been made use of, and no pain of any consequence has been felt up to this time. The articulation is perfect, the tooth is as firm as other molars in her mouth, and it is closed with a good temporary filling, which I shall in a short time replace with a permanent one of gold; and much to my own and the young lady's gratification, her courage bids fair to meet with what, in her estimation, is a fair compensation for her sufferings. The part of the root removed (which is shown) presented these characteristics; the periosteum was greatly inflamed, but just outside the apical foramen there was a small mass of apparent pulp tissue, having somewhat the appearance of an abscess, only smaller, and apparently solid in structure. This mass was placed beneath the field of a microscope, and was found to consist of true pulp tissue, containing multitudes of calcareous granules. Here, then was the secret of all my trouble; instead of a calcified pulp, as shown in these specimens, or nodules in the pulp, which many doubtless have seen, there was developed or deposited granular matter, which, so long as present, excited intense inflammation, first in the tooth pulp and periosteum, and lastly in the periosteum and tooth socket. The object in cutting off the root was, to allow a place for the accumulation of the effusion, which would certainly be poured out, as the result of the extraction, and which, accumulating, would tend to protrude the tooth from its socket, and interfere with articulation—a result which generally follows when teeth are replaced, and this action is neglected, causing the tooth sooner or later to drop from its socket, or be a constant source of annoyance to the patient. My belief is, that the remaining periosteum on the root of the tooth is at this time living; that that membrane does not undergo molecular death so readily as other structures, and that under favorable circumstances vitality will remain. I have proved this position beyond the shadow of a doubt, in cases where teeth have been returned to their sockets, and have been extracted, living periosteum being found upon them. That a separated pulp can unite to its former connection does not admit of a probability, for the retraction and contraction of the divided vessels would prevent such a result from being accomplished were union by adhesion possible; therefore, when a tooth is inserted, with the pulp remaining, there is every probability that alveolar abscess will occur, and the absence of discoloration is only an evidence that the absorbents have carried off the effete materials, the result of a disintegration of the pulp.

As a fitting close to this paper may we not assume that the transplanting of teeth opens to us a method of successfully treating some obscure dental diseases, and of combating intractable alveolar abscess, partial necrosis, exostosis, and perhaps other dental affections. It seems to me the answer must be in the affirmative.—*Dental Times.*

